# Comparing the cultivated cochlear cells derived from neonatal and adult mouse

**DOI:** 10.1186/1479-5876-12-150

**Published:** 2014-05-29

**Authors:** Xiangxin Lou, Youyi Dong, Jing Xie, Xianliu Wang, Liangliang Yang, Masaaki Tokuda, Yanzhong Zhang

**Affiliations:** 1 Department of Bioengineering, College of Chemistry, Chemical Engineering and Biotechnology, Donghua University, 2999 North Renmin Road, Shanghai 201620, China; 2 Department of Cell Physiology, Faculty of Medicine, Kagawa University, 1750-1 Ikenobe, Miki-cho, Kita-gun, Kagawa 761-0793, Japan

**Keywords:** Cochlea, Hair cell, Sphere, Progenitor cell

## Abstract

**Background:**

Previous reports showed the presence of limited numbers of stem cells in neonatal murine cochlear sensory epithelia and these cells are progressively lost during the postnatal development. The goal of this study was to investigate whether stem cells can be derived from mature mouse cochleae under suspension culture conditions, and to analyze the expression of the stem cell and inner ear progenitor cell markers in cells dissociated from neonatal and adult mouse organs of Corti.

**Methods:**

Organs of Corti were dissected from postnatal day 1 (P1) or postnatal day 60 (P60) mouse. The dissociated cells were cultivated under suspension cultures conditions. Reverse transcription-polymerase chain reaction (RT-PCR) and immunocytochemistry were conducted for phenotype characterization.

**Results:**

The number of cochlear stem cells (otospheres) yielded from P1 organ of Corti was significantly higher than that of the P60 organ of Corti. RT-PCR analyses showed that the stem markers, such as *nanog, sox2, klf4,* and *nestin* can be found to be distributed similarly in the cells derived from both of organisms, but the inner ear developmental/progenitor cell markers showed lower expression in P60 organ of Corti compared to P1. Immunocytochemistry results also revealed the evidence that P60 otospheres lacking of differentiation potential *in vitro*, which opposed to the strong differentiation potential of otospheres at P1 stage.

**Conclusions:**

Our findings suggest that the loss of numbers and features of stem cells in the adult organ of Corti is associated with the substantial down-regulation of inner ear progenitor key-markers during maturation of the cells in organ of Corti.

## Background

The mammalian inner ear has very limited ability to regenerate lost sensory hair cells [1,2]. While previous studies demonstrate that early postnatal cochlea harbors stem/progenitor-like cells and show a limited regenerative/repair capacity [3-13], and these properties, however, are progressively lost later during the postnatal development of the inner ear [5].

Despite the pluripotent differentiation potential of these cells, recovery does not occur to any significant extent after damage to hair cells in the adult mammalian cochlea [3]. Nevertheless, some reports show the presence of limited numbers of nestin-positive stem cells in adult mouse organ of Corti [5], suggesting that there may be some intrinsic potential for repairing hair cells. Therefore, it would be of great interest to explore whether there is the ability of sphere formation from cells harvested during later postnatal and mature stages.

It is well known that the adult mammalian cochleae lack regenerative ability and the irreversible degeneration of cochlear sensory hair cells leads to permanent hearing loss [14], but little is known about the genes and pathways that are potentially involved in this difference of the regenerative/repair potentialities between early postnatal and adult mammalian cochlear sensory epithelia (SE) [14]. The aims of this study are: 1) To investigate whether some remnants of regenerative ability of quiescent progenitor/stem cells can be isolated from mature cochlea, we compare the sphere-forming capabilities of cultured cells derived from P1 and P60 SE; 2) To explore changes of genes and pathways underlying the stem/progenitor cells maintenance and the capacity of regeneration/repair between P1 and P60 SE-derived cells. We examined the expression of a number of genes that were known as stem markers and early inner ear cell markers. These genes have been shown to be linked into known networks and pathways implicated in the mammalian cochlea. In addition, we investigate whether these cells can be differentiated into hair cells and supporting cells *in vitro* using immunocytochemistry.

## Materials and methods

### Animals

P1 and P60 C57/BL6 mouse pups (Slac laboratory animal, Shanghai, China) from different litters were used. Animals were housed with mothers in Animal House (College of Chemistry, Chemical Engineering and Biotechnology, Donghua University, China). During this study, animal care and use were in strict accordance with the animal welfare guidelines of the Helsinki Declaration.

### Cell culture procedure

**Figure 1:**
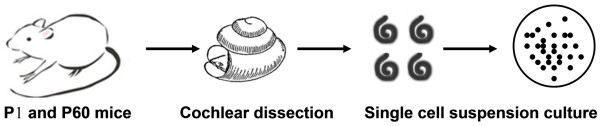
Tissue dissection and cell handling procedure.

Dissociated cell cultures were obtained under aseptic conditions from P1 and P60 mice as previously described [15] (Figure 1). In brief, SE sheets were isolated from cochleae in Hanks’ buffered salt solution (HBSS, Invitrogen) at 4°C, PH 7.4. Tissues were subjected to 0.125% trypsin in PBS solution (Invitrogen) for 15 min, at 37°C, then blocked by trypsin inhibitor and DNAse I solution (Sigma). After gently mechanical dissociation, the pellets were suspended in DMEM/F12 (Dulbecco’s Modified Eagle Medium: Nutrient Mixture F-12) 1:1 Mixture (Invitrogen) supplemented with N2 and B27 supplements (Invitrogen), EGF (20 ng/ml) (R&D Systems), bFGF (10 ng/ml) (Wako, Japan), IGF-1(50 ng/ml) (R&D Systems), ampicillin (50 ng/ml; Sigma) and heparin sulphate (50 ng/ml) (Sigma). The suspension was passed through a 70 μm cell strainer (BD Labware) into 6 well plastic Petri dishes (Greiner). Cell cultures were incubated under 37°C, 5% CO_2_, half of the medium was replaced every 2 days. At day 3, cell suspension was replated in new Petri dishes, the attached cells were abandoned. The suspending otospheres obtained from P1 or P60 organ of Corti were assessed in later experiments. For analysis of cell differentiation, we maintained the attached sphere-derived cells in a humidified incubator in a 5% CO_2_ at 37°C in differentiation medium consisting of DMEM/F12 mixed (1:1) supplemented with N2 and B27 (medium and supplements were from Invitrogen), 10% fetal bovine serum (Invitrogen), and ampicillin (50 ng/ml; Sigma). Half of the medium was replaced every 2 days. The differentiated cells were analyzed by immunofluorescence 7 days after plating.

### Cell yield and viability

The yield and cell viability were determined by using trypan blue vital staining. Four cochleae were dissected from P1 and P60 mice, respectively. The dissociated organ of corti-derived cells were seeded under suspension culture condition, 100 μl cell suspension of each condition was treated separately with 100 μl of 0.4% trypan blue. Using bright field optics, numbers of stained cells with intact plasmamembranes were determined.

Cell proliferation ability was evaluated by 3-(4, 5-dimethylthiazol-2-yl)-2, 5-diphenyltetrazolium bromide (MTT) solution (MTT assay kit, Sigma, USA). Briefly, the dissociated organ of Corti-derived cells were plated at 1000 cells/well in 96 well dishes. After the predetermined time points of incubation, the medium on these samples was removed and 10 μl of 5 mg/ml MTT solution was added and assayed according to the manufacturer’s instructions. Optical density of solutions in wells was measured at 570 nm using a photometer (MK3 Multilabel Plate Reader, Thermo, USA).

### RT-PCR assay

Total RNA was isolated from P1 or P60 mice SE and SE-derived otospheres respectively by using RNeasy Mini Kits (Qiagen), and a mouse embryonic stem cells (ESc) line, G4-2, was taken as positive control to show stem markers. We used 500 ng of total RNA from each group for reverse transcription (RT) by using Superscript III (Invitrogen). We determined the expression of mRNA of stem markers (*nanog, sox2, oct3/4, klf4, c-myc and nestin*), early inner ear cells markers (*jagged1, pax2, brn3.1, bmp7, myosin 7a* and *p27kip1*). Polymerase chain reaction (PCR) analysis was performed by using the following primers pairs: ***nanog*** (fw: AGG GTC TGC TAC TGA GAT GCT CTG, rv: CAA CCA CTG GTT TTT CTG CCA CCG); ***sox2*** (fw: TAG AGC TAG ACT CCG GGC GAT GA, rv: TTG CCT TAA ACA AGA CCA CGA AA); ***klf4*** (fw: CCA ACT TGA ACA TGC CCG GAC TT, rv: TCT GCT TAA AGG CAT ACT TGG GA); ***oct3/4*** (fw: TCT TTC CAC CAG GCC CCC GGC TC, rv: TGC GGG CGC ACA TGG GGA GAT CC); ***c-myc*** (fw: TGA CCT AAC TCG AGG AGG AGC TGG AAT C, rv: TTA TGC ACC AGA GTT TCG AAG CTG TTC G); ***nestin*** (fw: GAT CGC TCA GAT CCT GGA AG, rv: AGA GAA GGA TGT TGG GCT GA); ***jagged1*** (fw: GGT CCT GGA TGA CCA GTG TT, rv: GTT CGG TGG TAA GAC CTG GA); ***pax2*** (fw: CCC ACA TTA GAG GAG GTG GA, rv: GAC GCT CAA AGA CTC GAT CC); ***brn3.1*** (fw: GTC TCA GCG ATG TGG AGT CA, rv: TCA TGT TGT TGT GCG ACA GA); ***bmp7*** (fw: TCT TCC ACC CTC GAT ACC AC, rv: GCT GTC CAG CAA GAA GAG GT); ***myosin 7a*** (fw: CAC TGG ACA TGA TTG CCA AC, rv: ATT CCA AAC TGG GTC TCG TG); ***p27kip1*** (fw: ATT GGG TCT CAG GCA AAC TC, rv: TTC TGT TCT GTT GGC CCT TT); ***GAPDH*** (fw: GGG TGT GAA CCA CGA GAA AT, rv: ACA GTC TTC TGG GTG GCA GT). The RT-PCR experiments were independently repeated 3 times with the same total RNA material. Control RCR reactions were performed lacking cDNA (RT-).

### Immunochemistry procedure

To characterize the expression of stem and inner ear progenitor cell maker expression, the first propagation otospheres derived from P1 or P60 organs of Corti were selected for immunostaining analysis. The primary antibodies used were as follows, mouse monoclonal antibody for Oct3/4 (1:500, Santa Cruz Biotechnology, Santa Cruz, CA), for Nestin (1:500, BD Biosciences); goat polyclonal antibody for Sox2 (1:200, Santa Cruz Biotechnology); Pax2 (1:200, BD Biosciences) and for Jagged1 (1:200, Santa Cruz Biotechnology).

The differentiative abilities of P1 and P60 otospheres were characterized by immunocytochemistry using inner ear cell specific markers. After fixation in 4% paraformaldehyde, cells were washed with 0.1 M phosphate-buffered saline (PBS, PH 7.4) for 15 min. Nonspecific staining was blocked in PBS containing 0.2% Triton X-100 (Sigma) and 10% horse serum solution for 30 min. Primary antibodies Myosin7a (1:500, Proteus Biosciences, Ramona, CA), p27kip1 (1:200, NeoMarkers, Fremont, CA), and βIII-tubulin (1:500, Santa Cruz Biotechnology, Santa Cruz, CA) were applied overnight in PBS with 10% horse serum and 0.2% Triton X-100 at 4°C. After 3 times wash, FITC (fluorescein isothiocyanate) or TRITC (Tetramethyl Rhodamine) conjugated second antibodies (Invitrogen) were added for 60 min, counterstaining with DAPI (4′, 6-diamidino-2-phenylindole) (Invitrogen) was performed to visualize cell nuclei. Specimens were examined by confocal microscope (Leica). Negative control experiments were performed as above by omitting the primary antibodies.

### Statistical analysis

Quantitative data are expressed as the mean ± SD. The statistical process was examined by student’s unpaired two-tailed *t*-test. Significant differences are indicated by * (*p* < 0. 01).

## Results

### Cell viability and proliferation

**Figure 2:**
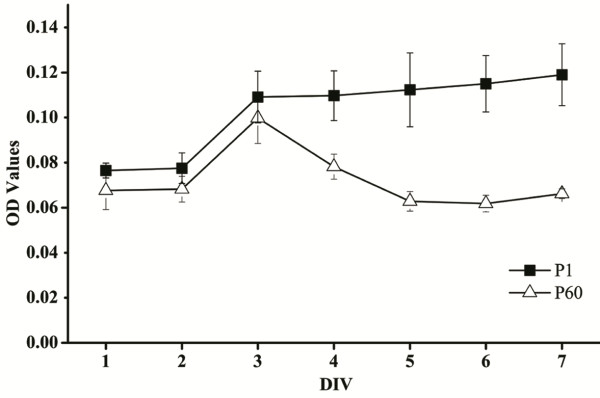
**MTT assays of the cell proliferation abilities of P1 and P60 organ of Corti-derived cells.** Data represent the cell proliferation at 1 to 7 days, and which is taken as a mean value of the triplicates (n = 6).

By staining with trypan blue, we observed that the viable cells from P1 organ of Corti 9.6 ± 0.9 (mean ± SD) × 10^4^ were nearly 2-fold higher compared to P60 5.4 ± 1.1 (mean ± SD) × 10^4^. MTT assay had been used to investigate rates of cell proliferation of P1 and P60 organ of Corti-derived cells. We observed that significantly enhanced proliferation of both cells from day 1 to 3, and maximum effect was produced at day 3 (Figure 2). P1 cells showed increasing in proliferation at all time points, while enhanced proliferation was not observed in P60 cells, which changed to reducing from day 3 to 5 and kept stably from day 5 to 7.

### Otospheres harvested from P1 and P60 organ of Corti

**Figure 3:**
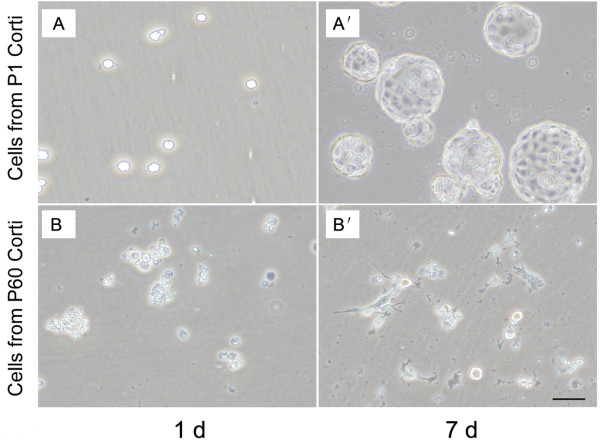
**Images represent the otospheres of culturing dissociated P1 or P60 organ of Corti. (A)** One day cultured cells dissociated from P1 organ of Corti; **(A**׳**)** Otospheres from suspension culture seven days of dissociated P1 organ of Corti; **(B)** One day culture of cells dissociated from P60 organ of Corti; **(B**׳**)** After seven days culture, only very few and small sphere cells were observed from dissociated P60 organ of Corti; Scale bar = 50 μm.

As shown in Figure 3, cells dissociated from P1 organ of Corti formed nice spheres, and they appeared to continue to proliferate during the course of culture at least 1 week (Figure 3A, A׳). While cells dissociated from P60 organ of Corti failed to form nice spheres during culture, only very few spheres could be found in several attempts we made (Figure 3B, B׳).

As for the otospheres number analysis, significant difference in sphere-forming cells number between P1 and P60 organ of Corti was observed in present study (Table 1). In contrast to P60 organ of Corti, the neonatal organ of Corti yielded much more sphere-forming cells, and this situation was maintained during the period of study, no any morphological sign of differentiation was observed. During propagation culture, we found otospheres from P1 could be passaged several propagations, but we could not acquire second generation of otospheres from P60.

**Table 1:** Otosphere numbers harvested from P1 and P60 cochleae

**Age**	**Number of otospheres (n = 5)**	**Dissected cochleae numbers**	**Treatment**
P1	3406 ± 245*	4	Suspension culture 7 days
P60	2 ± 1	4	Suspension culture 7 days

Data are mean ± SD. Significant differences are indicated by *(*p <* 0.01).

### Expression of stem and inner ear progenitor cell markers in SE and SE-derived otospheres harvested from P1 and P60 cochleae

**Figure 4:**
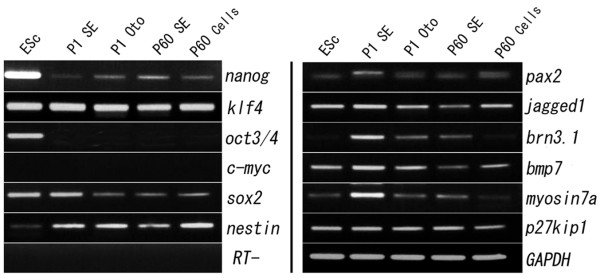
**RT-PCR analysis of the expression of stem cell and inner ear progenitor cell markers in the P1 and P60 SE, and the SE derived otospheres.** GAPDH expression analysis is shown as reference, and RT- is shown as negative control. ESc: embryonic stem cells; SE: sensory epithelia; Oto: otospheres.

The obvious reduction of the ability of cochlear organs from P60 mice to generate otospheres raises the question whether this loss of regenerative capacity is reflected in the expression levels of marker genes for stem cells and/or inner ear progenitor cells. We detected the expression of mRNA of stem markers, *nanog, sox2, klf4, nestin,* and early otic cell markers, *pax2, jagged1, brn3.1, bmp7, p27kip1, and myosin7a* (Figure 4). *Oct3/4* was found only expressed in ESc but not in otospheres. No *c-myc* expression was detected in this study. Indeed, the mRNA expression of all stem cell markers that we investigated was stably maintained in P1 and P60 SE as well as SE-derived otospheres (Figure 4)*.* However, we found inner ear developmental/progenitor markers, i.e. *pax2, jagged1, bmp7, brn3.1, p27kip1* and *myosin7a* showed lower expression in P60 SE and SE-derived otospheres compared with P1 (Figure 4).

### Immunocytochemistry results

**Figure 5:**
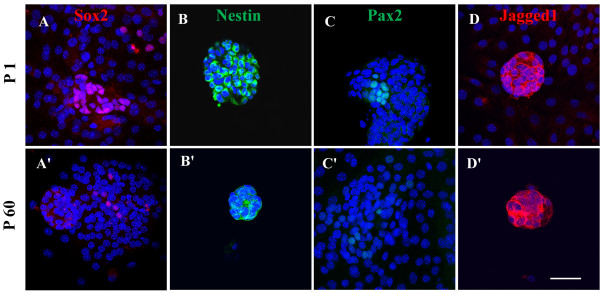
**Immunostaining for stem markers: Sox2 (A, A׳) and Nestin (B, B׳), and inner ear progenitor cell markers, Pax2 (C, C׳) and Jagged1 (D, D׳) in P1 and P60 otospheres, respectively.** DAPI identifies the cell nucleus in blue. Scale bar = 50 μm.

**Figure 6:**
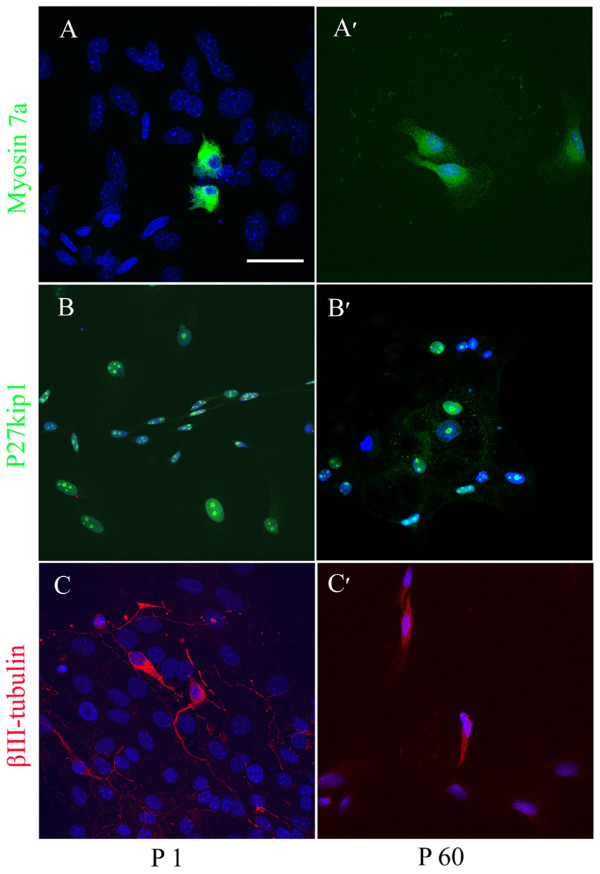
**Immunofluorescence of differentiated cells derived from P1 and P60 otospheres.** Myosin7a positive cells (green) were found in P1 **(A)** and P60 **(A׳)** differentiated otospheres; p27kip1 labels supporting cell nuclei, as shown in P1 **(B)** and P60 **(B׳)**; βIII-tubulin positive cells were observed from differentiated P1 **(C)** and P60 **(C׳)** otospheres. DAPI identifies the cell nucleus in blue. Scale bar = 50 μm.

Otospheres from P1 and P60 organs of Corti exhibited the expression of stem markers, Sox2 (Figure 5A, A׳) and Nestin (Figure 5B, B׳) as well as inner ear progenitors, Pax2 (Figure 5C, C׳) and Jagged1 (Figure 5D, D′), and no expression of Oct3/4 (data were no shown). As for P1 SE-derived otospheres, Sox2 was found 30 ± 3.4 (mean ± SD)% of total otospheres, Nestin was seen in 91 ± 12.4%, Pax2 was 18 ± 10.2% and Jagged1 was found in all otospheres. However, only very few otospheres from P60 organs of Cotri could be obtained.As for cell differentiation, our results showed the staining of protein markers for inner ear cells upon culturing of P1 and P60 otospheres in differentiation medium (Figure 6). All markers employed: Myosin7a (Figure 6A and A׳) for hair cells, p27kip1 (Figure 6B and B׳) for supporting cells, and βIII-tubulin (Figure 6C and C׳) for neural cells (myosin7a in cell processes, βIII-tubulin in the plasma membrane and neurite, and nuclear localization for p27kip1). This confirmed the undifferentiated phenotype of the two kinds of otospheres and their commitment to differentiate to different cell types of the inner ear. However, only very few immunostaining positive cells were observed in the differentiated cells from P60 otospheres. We deduced that the lack of demonstration of hair cell, neural cell and supporting cell differentiation from P60 otospheres is most probably due to their limited cell number. The other reason may also be related to the relatively maturation of P60 cochlea compared with P1.

## Discussion

Hearing loss is often caused by loss of hair cells (HCs) due to a variety of factors that generate oxidative stress including noise, ototoxic drugs, cisplatin or aging, etc. [2,16]. The primary cause of hearing loss is the damage or death of the sensory cells in the auditory part of the inner ear, i.e. the cochlea. In mammals, HCs are produced only during embryonic development and do not regenerate, if these cells are lost during postnatal and adult life, the result is profound sensorineural deafness [17]. The adult mammalian cochlea lacks regenerative ability and the irreversible degeneration of cochlear sensory hair cells leads to permanent hearing loss [17]. The previous studies show that early postnatal cochleae harbor stem/progenitor-like cells and show a limited regenerative/repair capacity [4,7-15,17,18], however, these properties are progressively lost later during the postnatal development [5]. Furthermore, the discovery of stem cells in the adult mouse utricular SE spurred great interest to investigate whether other parts of the inner ear also harbor stem cells that can be isolated by *in vitro* culture [19]. In contrast to adult mammalian vestibular SE, postnatal mouse organ of Corti has very limited capabilities to form sphere cells [5]. Little is known about the genes and pathways that are potentially involved in this difference of the regenerative/repair potentialities between early postnatal and adult mammalian cochlear SE.

To investigate whether some remnants of regenerative ability of quiescent progenitor/stem cells can be isolated from mature cochlea, we compare the cell viability as well as sphere-forming capabilities of cultured cells derived from P1 and P60 organs of Corti, respectively. A notable observation was that the cell viability of P1 organ of Corti-derived cells were 2-fold higher than P60, indicating the cell populations have a wide-ranging alterations at different ages. MTT assay demonstrated that P1 cells showed marked enhancement of proliferative activity during expansion culture *in vitro*, which suggested that P1 cells have higher potential for proliferation compared to P60 cells.

As for the sphere-forming capabilities, we only observed very few sphere formation (2 ± 1, n = 5) from four dissociated P60 SE, which can be negligible compared to P1 tissues (3406 ± 245, n = 5), indicating that the stem cells of the auditory system disappear during the mature stage. Our result is consistent with the data of Oshima’s report that the total otosphere numbers are indeed decreased from P21 to P42 organ of Corti and spiral ganglion [5]. Nevertheless, no further data explains the mass loss of sphere formation in the adult organ of Corti. We first reason that the observed loss of sphere formation is reflected the cell number loss during mature. However, when we seeded the same number of cells derived from P1 or P60 cochleae, respectively, we still found that the stark loss of capacity for sphere formation in P60 stage. As for the propagation culture, we found otospheres from P1 can be passaged for several propagations and showed high self-renewal ability and viability. Wherein, we could not acquire second propagation of sphere-forming cells from dissociated primary ototspheres at P60. The reason may be that the self-renewal capacity of the limited numbers of stem cells in adult organ of Corti is progressively lost during the postnatal development. We hypothesize that as an alternative to the loss of stem cells might be interpreted as a loss of stem cell features. The hypothesis is further confirmed by the analysis of RT-PCR expression using stem cell and inner ear progenitor cell markers. The diminishing stem cell features at P60 cochlear cells can provide a reasonable explanation for the long known regenerative inability of the maturing cochlea [20-24]. This view is supported by our observation that the expression of mRNA of inner ear developmental/progenitor became lower from P1 to P60. Future study will perform real-time PCR or western-blotting experiments to complement the quantitative data. We hypothesize that the loss of stem cells or the loss of stem cell features in the adult organ of Corti is accompanied by a substantial reduction at the expression level of inner ear progenitor cell markers. Whereas the sustained expression of stem cell markers between P1 and P60 tissues is an indication of the maintenance of stemness in mammalian SE, but it is still know little about how to stimulate these quiescent stem cells to self-renewal and proliferate in maturing stage [25].

C57/BL6 is most widely used inbred strain in hearing research. The C57/BL6 mice exhibit a high frequency hearing loss by 3–6 months of age that progresses to a profound impairment by 15 months. This strain has a known age associated hearing loss due to cdh23ahl [26]. Because of the possibility of potential influence of this in ours studies, we examined the otospheres from P 60 mice. We have performed the experiments on ICR mice, and found the expression of stem cell and inner ear progenitor cell markers are associated with age (data were not shown). Because of the possibility of strain differences, it would be great interest to examine the cochlear stem cell phenotypes from different strains, i.e. ICR, BALB/c and C57/BL6 mice in future study.

The results of this approach may provide directions for future investigations into the understanding of the known difference in the ability for regeneration/repair between the early postnatal/developing and adult cochleae. In addition, it would be interesting in future studies to remove or reduce the potential mechanisms of repression to active these stem cells [27], and it is worth pursuing the best growth factor combination that potentially leads to increased cell survival, proliferation and differentiation. Other method, such as gene transfer [28] or iPS cell technology [29,30] can be performed to achieve the goal.

## Conclusion

Sphere-forming stem cells from the mouse inner ear are an important tool for the development of cellular replacement strategies of damaged inner ears and are a bona fide progenitor cell source for transplantation studies. Dissociated P60 organ of Corti produced very few otospheres *in vitro*, expressing stem markers, such as sox2 and nestin similarly to P1 SE and otospheres. However, inner ear developmental/progenitor cell show lower expression in P60 stage compared with to P1, which may be contribute the reducing sphere-forming ability from P60 SE. And the lower number of cells for P60 otospheres may relate to its lack of differentiation potential *in vitro*, as opposed to the strong differentiation potential observed *in vitro* for P1 otospheres. However, the limited sphere cell number and restricted differentiation potential observed by us at P60 organ of Corti reinforce this organism as potential experimental studies to search for the mechanisms for organ of Corti regeneration in mammalian cochlea.

## Competing interests

The authors declare that they have no competing interests.

## Authors’ contributions

XXL, JX, XLW and LLY carried out the tissue dissection, cell culture and data collection. JX, XLW and LLY carried out the immunoassay and RT-PCR. XXL performed the analysis and interpretation of data. XXL, YYD and MT were involved in drafting part of the manuscript. YZZ contributed the whole study and participated in the design and coordination of this project as well as manuscript writing. All authors reviewed and approved the final manuscript.
